# Exploring the mediating role of social environment in the relationship between built environment and mental health of older adults—evidence from Guangzhou, China

**DOI:** 10.3389/fpubh.2026.1706431

**Published:** 2026-01-26

**Authors:** Zhang Rongrong, Yang Yulu, Sun Yanan, He Xiong, Zhang Shaoyang, Han Jiaxiang, Cao Yongwang

**Affiliations:** 1 School of Political Science and Law, Zhengzhou University of Light Industry, Zhengzhou, China; 2 School of Geography and Planning, Sun Yat-Sen University, Guangzhou, China; 3 Guangzhou Academy of Social Sciences, Guangzhou, China

**Keywords:** older adults, mental health, built environment, social environment, China

## Abstract

**Introduction:**

The importance of social environment for the mental health of older adults is gaining increasing attention, while the mediating role of social environment has not yet been thoroughly investigated.

**Methods:**

Using point-of-interest (POI) data from Baidu and data on 20 communities in Guangzhou, China, which were collected through a questionnaire survey, this study employed a multi-level mediation model to investigate the relationship between the built environment and the mental health of older adults and to test the mediating effect of the social environment.

**Results:**

The findings indicated that park accessibility, as well as the distance to the nearest park are significantly positively correlated with the mental health of older adults, and the distance to the nearest public transit station is significantly negatively correlated. Social capital and community safety play significant mediating roles between the built environment and the mental health of older adults. Furthermore, the impact of built environment on mental health of older adults through the social environment, as well as the mediating pathways involved, exhibits significant differences across older adults with varying income levels.

**Discussion:**

This approach deepens the understanding of the pathways through which community environments impact the health of older adults in the Chinese context. The results provide a reference for policymakers and urban planners in planning healthy communities, age-friendly communities, and advancing an active aging society.

## 1 Introduction

Like many other countries, China has also entered an aging society, with its aging process accelerating at a rapid pace toward a deeply aged society (1). According to the seventh national census, in 2020 China’s population aged 60 and above reached 264.02 million, while those aged 65 and above numbered 190.64 million, accounting for 18.70 and 13.50% of the total population, respectively. Compared with the sixth national census results, the proportions of population aged 60 + and 65 + have increased by 5.44 and 4.63 percentage points, indicating a further deepening of population aging (2, 3). As China’s population aging continues to deepen, building a healthy aging society has become a key issue in social development and has attracted research attention from scholars (4–6). However, the overall health status of older adults in China is not optimistic, with mental health issues becoming increasingly prominent. According to the National Mental Health Development Report of China (2019–2020), nearly one-third of Chinese older adults experience psychological disorders. Consequently, addressing elderly mental health concerns is vital for China’s ongoing efforts to establish a healthy aging society.

Older adults are more likely to experience health-related changes and challenges as age advances, making them potentially more sensitive and susceptible to the residential environment than other age groups (7–9). Experts and scholars from various fields, such as planning, medicine, sociology, and geography, have begun to consider the importance of the community environment with regard to the mental health of older adults (10–12). Early research on the relationship between community environment and the mental health of older adults mainly focused on the built environment of the community, and was mostly conducted in developed Western countries. For example, a study on New York residents has indicated that people who live in a lower-quality built environment are more likely to suffer from depression (13). Putrik et al. (14) found that residents living close to the railway were exposed to a high number of stressors and reported worse mental health. In recent years, an increasing number of studies conducted in developing countries have also confirmed a significant correlation between the built environment of communities and the mental health of the older adults (15, 16). For example, the construction of community sports and entertainment facilities can effectively prevent mental health problems among the elderly (17). The built environment also provides opportunities for the cultivation of the social environment (18–21). When the community has a dense population, high land use mix, and dense road network, residents are more likely to participate in social interaction, and thus more likely to obtain opportunities to create mutual connections and social support (20). The leisure and entertainment facilities within walking distance of residential areas also provide a place for residents to socialize with their neighbors (21). This intentional or unintentional social interaction between neighbors can cultivate a sense of trust and reciprocity within the community, thereby fostering a sense of responsibility and cohesion (22). The chance encounters and formal meetings facilitated by the built environment can also enhance residents’ social capital. In addition to community facilities such as parks and trails that help cultivate social capital, the existence of libraries and community activity centers can also enhance social capital by increasing interaction opportunities and providing collective activity venues (23). The lack of public spaces in the community can hinder residents’ interaction and participation in social activities, leading to a stronger sense of insecurity and fear toward the community (24). The social environment, as perceived by individuals as social cohesion/support, collective efficacy, and neighborhood relationships, has gradually been recognized by scholars as having a significant impact on mental health. For example, community cohesion has been found to have a significant promoting effect on the mental health of older adults (25, 26). Community cohesion can enhance mutual trust and solidarity among neighbors (26), and improve mental health and well-being by promoting social and physical activity, mitigating the negative effects of stress (25, 27). Social support provides physiological and psychological advantages for people facing stressful physiological and psychological events, and is considered an important factor in reducing psychological distress when facing stressful events (28). Previous studies have confirmed the relationship between social support and mental health, mainly focusing on the protective (positive) role of social support in depression symptoms and happiness (29, 30), while social support also affects individuals’ health behaviors and physiological functions (29). Social capital is another important determinant of mental health (31). Social capital can affect mental health through various pathways such as collective socialization, informal social control, and collective efficacy (32). Community safety has also been found to maintain a significant positive correlation with mental health after adjusting for other individual and community factors (33, 34). The disorderly and chaotic environment of the community can trigger a sense of helplessness and fear among residents, directly or indirectly exacerbating their painful experiences and psychological pressure (35, 36). Considering that the community environment includes both the “hard” built environment and the “soft” social environment, the two are interdependent and work together to affect the health of residents as a complete functional unit. Scholars have begun to attempt to quantitatively analyze the built environment, social environment, and individual health within the same framework. Qiu et al. (37) set both the built environment and social environment as explanatory variables to identify environmental factors that affect residents’ mental health. Some scholars have also set some elements in the social environment as mediating variables and used structural equation modeling to explore whether the built environment affects individual health by acting on the social environment. Tao et al. (38) used a multi-level structural equation model based on survey data from Beijing in 2017 to confirm the complex relationship between built environment, social interaction, and mental health.

Building on the above insights, we found that although empirical research has focused on the unity and integrity of the environment, the mediating role of the social environment in the relationship between built environment and mental health still needs further exploration, and a more comprehensive consideration of multiple dimensions of the social environment is needed, rather than just focusing on one aspect. In addition, the residential self selection effect is also one of the focuses of academic attention. Currently, scholars are mostly concerned about the impact of residential self selection on the correlation between “built environment - activities.” For example, residents who already enjoy walking may choose to live in pedestrian friendly communities on their own (39). If the residential self selection effect is not controlled, it may overestimate the impact of the built environment on travel behavior, resulting in estimation bias (40). Similarly, the impact of the built environment on individual health may also be overestimated due to residential self selection (41). Due to the existence of residential self selection mechanisms, residents may choose their living environment based on their own preferences, which may interfere with the impact of the built environment on their mental health in the community (42). However, there are relatively few empirical studies considering self selection interference in the current research on the association between built environment and mental health, and further empirical testing is needed. This study is based on POI data and questionnaire survey data, with older adults from 20 communities in Guangzhou as research subjects, to explore the relationship between built environment and the mental health of older adults, focusing on the mediating role of social environment between the two, and controlling for estimation bias caused by residential self selection effects.

## 2 Methods

### 2.1 Data sources

As one of China’s megacities, Guangzhou has experienced a rapid increase in its elderly population. By the end of 2019, the number of people aged 60 and above reached 1.7551 million, accounting for 18.40% of the registered population. A multistage stratified probability proportionate to population size sampling technique (PPS), which enabled each unit to have the probability of being selected in proportion to its size (43), was adopted to select respondents.

**Figure 1:**
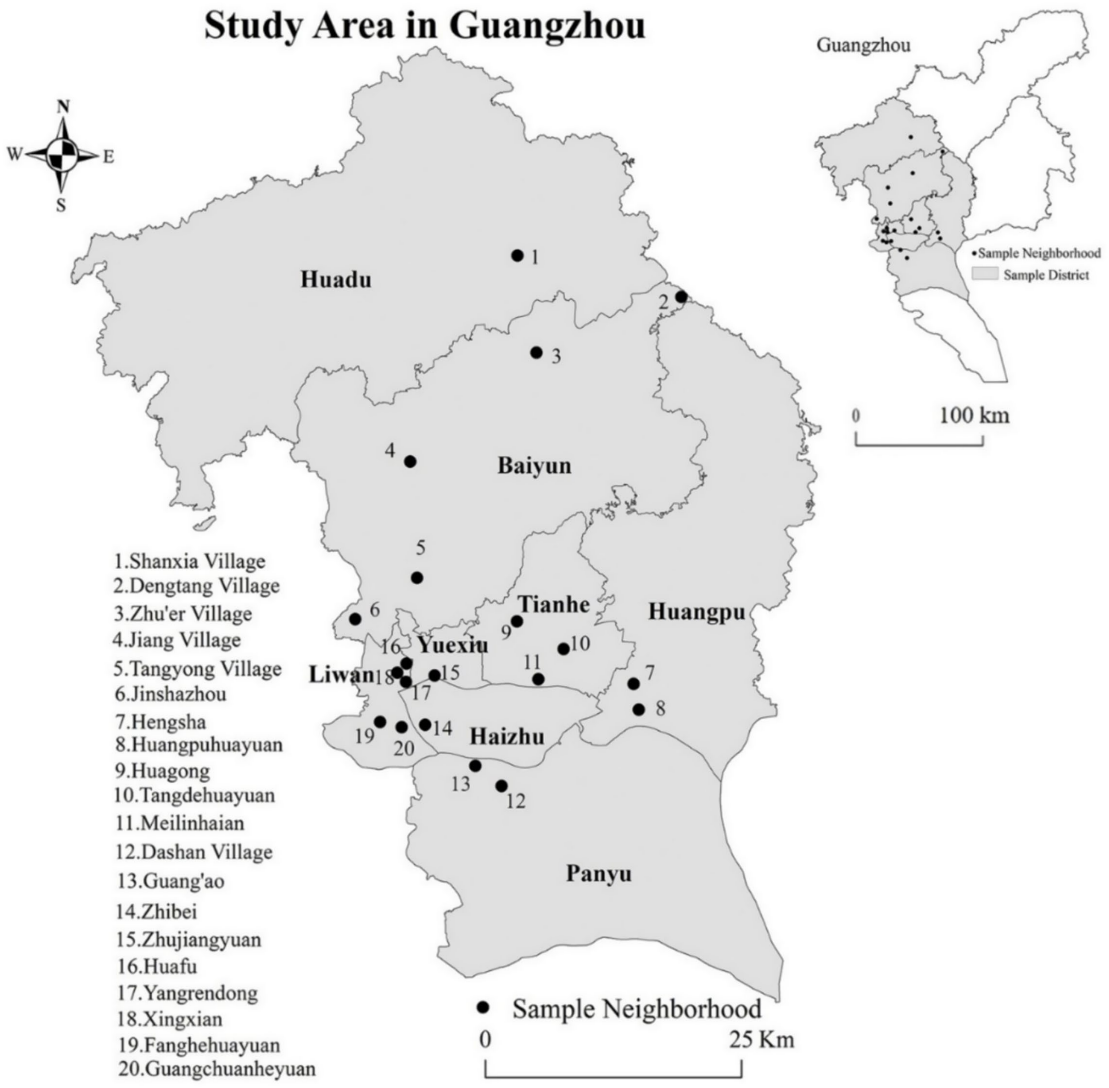
Location of 20 surveyed communities. Reproduced from “Location of surveyed communities” by Rongrong Zhang, Xiong He, Ying Liu, Ming Li and Chunshan Zhou, licensed under CC BY 4.0. Map from the National Natural Resources and Geospatial Basic Information Database (https://www.sgic.net.cn/web/geo/index.html#/Home).

First, based on previous research findings (44), we scientifically categorize Guangzhou’s social aging areas into six distinct types (Table 1). Building on this classification, we then select 18 representative streets from these six social areas, focusing on those with high factor scores and significant eigenvalues. During street selection, we prioritize two key principles: (1) selecting streets where the elderly population exceeds 10% and ranks highest, and (2) ensuring the chosen communities cover diverse housing types including commercial housing, institutional housing, rural self-built housing, affordable housing, old neighborhoods, and urban villages. Through this rigorous selection process, we ultimately identify 20 most representative case communities for subsequent random sampling surveys (Figure 1).

**Table 1:** Surveyed communities.

Community type	District	Subdistrict	Community	Housing type	Questionnaires collected
High-concentration area of older adults in an old urban area	Liwan	Hualin	Xingxian	Historical	25
		Longjin	Huafu	Historical	10
		Lingnan	Yangrendong	Historical	28
	Yuexiu	Zhuguang	Zhujiangyuan	Historical	68
Gathering areas for older adults who have retired from government enterprises and institutions	Liwan	Baihedong	Guangchuanheyuan	Danwei	108
	Haizhu	Nanshitou	Zhibei	Danwei	126
	Huangpu	Huangpu	Huangpu Garden	Commercial housing	29
	Tianhe	Yuancun Subdistrict	Meilinhaian	Commercial housing	36
Scattered distribution area of older adults who have retired from educational and scientific research institutions	Tianhe	Wushan	Huagong	Danwei	87
Mixed Population distribution areas	Liwan	Dongjiao	Fanghe Garden	Affordable housing	22
	Baiyun	Jinsha	Jinshazhou	Affordable housing	90
	Panyu	Luopu	Guang’ao	Commercial housing	17
	Huangpu	Dasha	Hengsha	Commercial housing	29
Concentrated distribution area of rural older adult population	Baiyun	Zhongluotan	Dengtang	Rural village	52
	Baiyun	Zhuyuan	Zhu’er	Rural village	32
	Baiyun	Jianggao	Jiangcun	Rural village	19
	Huadu	Huadong	Shanxia	Rural village	47
New development areas with young population	Baiyun	Xinshi	Tangchong	Urban villages	44
	Panyu	Dashi	Dashan	Urban villages	55
	Tianhe	Tangxia	Tangdehuayuan	Affordable housing	8

Second, we determine the exact number of questionnaires for each community using stratified proportional sampling based on permanent resident population data from the Sixth National Population Census, while selecting specific respondents through random sampling. We finally enrolled 1,000 study participants and a total of 932 valid questionnaires were completed, the completion rate was 93.2%. This study involving human participants were reviewed and approved by School of Geography and Planning, Sun Yat-sen University Ethics committee. All participants were informed about the aim of the survey, the selection criterion of the sample, and the assurance that the information was only used for research. The participants provided their oral informed consent to participate in this study.

### 2.2 Variables

#### 2.2.1 Dependent variable

This study uses the SF-36 scale (45), widely applied in health research, to measure mental health levels. The scale includes two dimensions - mental health and vitality - assessed through seven questions about respondents’ psychological feelings over the past 4 weeks. Specifically, these questions are: “Do you feel peaceful?,” “Do you feel happy?,” “Are you able to concentrate on your work,” “Have you been free from stress?,” “Have you been free from nervousness?,” “Have you been free from depression?,” and “Do you feel energetic?.” For measurement approach, each item is scored using a 5-point Likert scale (1means strongly disagree, 5 means strongly agree). Since existing studies demonstrate this variable follows approximately normal distribution (46), and in line with prior research (16, 46, 47), we treat the total mental health score as a continuous variable. The composite score is calculated by summing all item scores, with total scores ranging from 7 (poorest mental health status) to 35 (optimal mental health status). Notably, the SF-36 scale has demonstrated good validity and reliability in prior research (48, 49). The mental health scale in this study demonstrates excellent internal consistency, with a Cronbach’s a coefficient of 0.923.

#### 2.2.2 Independent variables

Considering that older adults are more active within their communities and utilize surrounding urban public facilities more frequently (50), the scope of the built environment selected for this study was the built environment surrounding the residential communities of older adults. However, the “community” used in this study was not an administrative community but an area defined by a 1 km buffer zone centered on the location of the community residents’ committee. The basis for delineating a 1 km buffer zone centered around the community neighborhood committee is as follows: (1) 1 km is a widely accepted daily activity radius that is friendly to the majority of residents, especially the older adults. It ensures that older adults will not give up when they go to the committee to handle affairs, participate in activities, or seek help due to the distance, so even if the community committee is not the geographical center of the community, it is still within the daily activity radius of older adults. (2) The community committee is the main gathering point of community resources, and community public service facilities (such as community service stations, cultural activity rooms, home-based elderly care centers, etc.) often rely on or are located near the committee layout, forming a small service complex. Defining an area around it can effectively evaluate service coverage. (3) The community committee is the social and psychological center of residents, and often becomes the “discussion center” and “problem-solving center” in the hearts of residents during long-term work. Its location may have a stronger sense of community identity due to history, transportation, or landmarks, playing an important role in creating the social environment of the community (community safety, community cohesion, etc.), even if it is not geographically centered.

Current measurements of the built environment are mostly based on the “5D” research dimensions proposed by Ewing and Cervero (51): Density, Diversity, Design, Destination accessibility, and Distance to transit. Considering previous research findings (52, 53) and data availability, this study selected “4D” (Density, Diversity, Destination accessibility, Distance to transit) to measure the community built environment (Table 2).

**Table 2:** Built environment of the community.

Type	Name of variables	Definition
Density	Population density	Population divided by the subdistrict area
Diversity	Mixed land use	Mixed degree of POI within the 1-km buffer
Accessibility	Facility accessibility	Number of POI within the 1-km buffer (mainly including 7 categories: catering service facilities, living service facilities, public sports facilities, shopping service facilities, cultural service facilities, government agencies, and medical service facilities)
	Park accessibility	Number of parks within the 1-km buffer
	Public transit station accessibility	Number of public transit stations within 1-km buffer
Distance to destination	Distance to the nearest park	–
	Distance to the nearest public transit station	–

#### 2.2.3 Mediators

The community social environment is the mediator in this study, primarily including four elements: social capital, social support, community cohesion, and community safety.

##### 2.2.3.1 Social capital

Social capital was measured via selected questions from the questionnaire that had been applied in empirical study in China (54). Personal belonging was measured with “I belong to this community.” Community trust was measured with “Do you trust the community residents’ committee/village committee?” and “I am satisfied with the overall environment of the community.” Community network was measured with “I know many people in the community” and “I have many relatives (besides children and spouse) with whom I have good relationships.” Based on a Likert scale, questions had 5 options, assigned values from 1 (strongly disagree) to 5 (strongly agree).

##### 2.2.3.2 Community cohesion

Following previous studies on community cohesion (5), we adopted a single-indicator measurement approach by directly asking respondents about their subjective perception of cohesion in their community, using the question “The cohesion in my community is strong.” Based on a Likert scale, the question had 5 options, assigned values from 1 (strongly disagree) to 5 (strongly agree).

##### 2.2.3.3 Social support

Based on previous research (55), we selected family support and community support variables to measure the social support received by older adults. Family support included emotional support and economic support, measured with “When you talk to your children about your worries or difficulties, do you feel they are willing to listen?” and “Have your children given you money or food to support your life?.” Answers were assigned 0 (No) and 1 (Yes). Community support was measured with “When you encounter difficulties, do you hope to get help from the community?” and “When I encounter difficulties, I can get help from the community.” Based on a Likert scale, these two questions had 5 options, assigned values from 1 (strongly disagree) to 5 (strongly agree).

##### 2.2.3.4 Community safety

Referring to questions on community safety designed in existing studies (34, 56), we used the question “I feel safe walking alone in the community during the day and night” to measure community safety. Answers had 5 options, assigned values from 1 (strongly disagree) to 5 (strongly agree).

#### 2.2.4 Control variables

To eliminate the influence of self-selection mechanisms, the control variables selected were the socio-economic attributes and individual preferences of the older adults. Socio-economic attributes included age, gender, marital status, education level, income, and living arrangement. Individual preferences included daily travel mode, smoking, and drinking.

### 2.3 Methods

#### 2.3.1 Multi-level linear regression

In this study, older adults are nested within specific communities. Communities provide the built and social environment for the older adults, but environmental differences between communities are significant. Therefore, the data structure is suitable for multi-level modeling. The socio-economic attributes of older adult respondents are treated as Level 1 variables, while the environmental attributes of the 20 communities they belong to are treated as Level 2 variables. Using Stata 16.0 software, a random intercept model was employed for data processing. The formula for the multi-level linear regression model is:


$$Y_{\mathrm{ij}}=\alpha +\beta X_{\mathrm{ij}}+\gamma Z_{j}+\mu_{j}+\varepsilon_{\mathrm{ij}}$$

Among them, individual i (1–932) is nested within community unit j (1–20). Yij represents the mental health level of the older adults i in community j; Xij denotes the individual-level socioeconomic attribute variables of the older adults i in community j; Zj stands for the community-level environmental attribute variables of community j; *α* is the intercept; $\;\mu_{j}$ is the community-level residual; and $\varepsilon_{\mathrm{ij}}\;$ is the individual-level residual.

This study employs the three-stage stepwise regression method and Bootstrap method to test the mediating effect of the mediating variable (57).

#### 2.3.2 Robustness test

The robustness test examines the robustness of the explanatory power of evaluation methods and indicators, that is, whether the evaluation methods and indicators still maintain a relatively consistent and stable interpretation of the evaluation results when certain parameters are changed (58). The commonly used methods for robustness testing include variable replacement method (59), sample size change method (60), sub sample regression method (61), supplementary variable method (62), etc. In this study, we chose the sub sample regression method and divided the total sample into two sub samples with and without relocation experience based on whether there was relocation experience, and conducted regression analysis separately. The rational choice theory holds that relocation is a rational choice made by older adults as actors under the influence of external situations, taking into account various factors such as individuals and families. At the individual level, older adults meet their economic and health needs through relocation. In terms of health needs, some older adults obtain higher quality medical resources or older adults care services through relocation to maintain and improve their health (63). Research has found that older adults with poor health conditions (64), experience of falls (65), and care needs (66) have a higher probability of relocating and tend to move to aging friendly environments or closer to medical institutions. Therefore, relocation can to some extent represent the housing choice behavior of older adults.

## 3 Results

### 3.1 Descriptive statistics

Table 3 demonstrates the profile of the respondents. The majority of respondents (75.429%) were aged 60–75. The proportion of men and women was almost equal. The highest proportion of respondents had a primary school education or below (41.416%), while the smallest proportion had a bachelor’s degree or above (2.682%). The average income of respondents was approximately 3,000 RMB. About 60% of respondents lived with their children, while about 40% lived alone or with a spouse. Over 70% of older adults preferred walking or cycling for daily travel. The average mental health score was 31.710. The average population density in the surveyed communities is 19,440 people/km^2^, with a mean functional mix of 0.667. Within the 1 km buffer zone surrounding the communities, the average number of public facilities is 4,044.568, the average number of parks is 4.734, and the average number of public transit station is 28.733. The average distance from the communities to the nearest park is 0.482 km, and to the nearest public transit station is 0.267 km. The average scores for social capital, social support, community cohesion, and community safety were 19.159, 13.503, 3.820, and 3.961, respectively.

**Table 3:** Summary statistics for all variables.

Variables	Mean (SD)/*N* (%)
Dependent variable	
Mental health	31.710(4.950)
Independent variable	
Population density	1.944(1.824)
Mixed land use	0.667 (0.085)
Facility accessibility	4044.568(3546.420)
Park accessibility	4.734 (4.519)
Public transit station accessibility	28.733 (16.137)
Distance to the nearest park	0.482 (0.578)
Distance to the nearest public transit station	0.267 (0.225)
Mediating variable	
Social capital	19.159 (2.439)
Social support	13.503 (2.433)
Community cohesion	3.820 (0.842)
Community safety	3.961 (0.831)
Individual variable	
Age (year)	
60–75	703 (75.429%)
Above 75	229 (24.571%)
Gender	
Male	405 (43.455%)
Female	527 (56.545%)
Educational level	
Primary school and below	386 (41.416%)
Junior middle school	265 (28.433%)
High school or technical secondary school	218 (23.391%)
Training school	38 (4.077%)
Bachelor’s degree or above	25 (2.682%)
Marital status	
Unmarried, widowed, or divorced	215 (23.069%)
Married	717 (76.931%)
Living arrangement	
Living alone or with a spouse	389 (41.738%)
Living with other family members	543 (58.262%)
Monthly income [mean (SD)]	3183.5 (2568.919)
Travel preferences	
Walk or ride	(72.841%)
Public transport	(3.750%)
Drive or take taxis	(23.409%)

### 3.2 Relationship between built environment and mental health

To test the applicability of the multi-level mediation model, the Intraclass Correlation Coefficient (ICC) for the null model of older adults’ mental health was first calculated. The ICC value was 0.113, indicating that 11.30% of the total variance in mental health levels was explained by differences between communities. Therefore, a multi-level regression model was appropriate. Stata 16.0 was then used to analyze the impact of the built environment on the mental health of older adults. Table 4 shows the results of the multi-level linear regression. Model 1 is the benchmark model, including only the built environment and individual socio-economic attributes.

**Table 4:** Associations between the built environment and mental health of older adults (possible mediators).

Variables	Modle1 (DV: Mental health)		Modle1a (DV: Social capital)		Modle1b (DV: Social support)		Modle1c (DV: Community cohesion)		Modle1d (DV: Community safety)	
	Coef.	S. E	Coef.	S. E	Coef.	S. E	Coef.	S. E	Coef.	S. E
Built environment										
Population density	−0.035	0.197	0.097	0.179	−0.131	0.119	0.043	0.059	0.051	0.035
Mixed land use	−4.018	3.427	−0.634	3.631	−0.684	2.191	−0.689	1.190	2.278***	0.619
Facility accessibility	<−0.001	<0.001	<0.001*	<0.001	<−0.001	<0.001	<−0.001	1.190	<0.001**	<0.001
Park accessibility	0.058**	0.027	0.157	0.061	0.113**	0.037	−0.002	0.020	−0.109	0.010
Public transit station accessibility	0.037	0.059	−0.018	0.027	0.026	0.017	0.006	0.009	−0.013**	0.005
Distance to the nearest park	2.764**	1.289	0.764	0.527	0.299	0.317	0.264	0.173	0.226**	0.088
Distance to the nearest public transit station	−1.157**	0.507	−3.284**	1.506	−0.072	0.868	−0.778	0.493	−0.255	0.233
Individual variable										
Age (ref.60–75)	−0.628	0.406	−0.114	0.192	0.124	0.201	0.065	0.069	0.022	0.068
Gender (ref. female)	0.596*	0.326	−0.512**	0.165	−0.249	0.172	−0.219***	0.059	−0.067	0.058
Marital status (ref. unmarried)										
Widowed or divorced	1.195	1.582	−0.333	0.822	1.390	0.865	0.124	0.294	0.474	0.289
Married	1.270	1.545	−0.860	0.804	0.943	0.846	0.125	0.288	0.459	0.283
Education level (ref. primary school and below)										
Junior high school	−0.512	0.409	−0.408*	0.201	−0.252	0.211	−0.093	0.072	−0.173**	0.070
Senior high school or technical secondary school	−0.561	0.448	−0.158	0.227	0.078	0.238	−0.124	0.081	−0.133*	0.080
Junior college	0.078	0.831	−0.408	0.405	−0.319	0.424	−0.448**	0.145	−0.153	0.142
University and above	−1.082	1.065	−0.438	0.524	−0.079	0.551	−0.136	0.187	0.216	0.184
Monthly Income	−0.725***	0.174	0.565***	0.092	0.369***	0.095	0.101**	0.033	−0.011	0.031
Living Arrangement (ref. living alone or with a spouse)										
Living with children	−0.635*	0.324	−0.126	0.163	−0.059	0.170	−0.053	0.058	−0.062	0.057
Travel preferences (ref. walking or cycling)										
Public transportation	0.112	0.877	0.882**	0.438	0.045	0.460	0.167	0.157	−0.030	0.154
Self-driving or taking a taxi	−0.458	0.380	0.386**	0.193	0.087	0.202	0.051	0.069	−0.173**	0.067
Constant	19.203***	2.955	17.189***	2.722	9.649***	1.775	3.582***	0.899	2.232***	0.523
Log likelihood	−2587.941		−1924.860		−1960.336		−1036.944		−1010.926	
Prob > chi2	0.000		0.000		0.002		0.000		0.000	
AIC	5325.092		3893.709		3964.672		2117.888		2065.852	

***, **, * significant at the 1, 5, and 10% threshold levels, respectively.

As for the built environment, park accessibility (*β* = 0.058, *p* < 0.05) and distance to the nearest park (*β* = 2.764, *p* < 0.05) were significantly positively correlated with the mental health of older adults. The distance to the nearest public transit station (*β* = −1.157, *p* < 0.05) was significantly negatively correlated with the mental health of older adults. Population density, mixed land use, facility accessibility, and public transit accessibility showed no significant correlation with the mental health of older adults.

Regarding individual socio-economic attributes, the results indicated that compared to females, male older adults were more likely to have good mental health (*β* = 0.596, *p* < 0.1). Older adults with higher income levels were more likely to have good mental health (*β* = 0.725, *p* < 0.01). Compared to living alone, older adults living with their children had relatively poorer mental health (*β* = −0.635, *p* < 0.1).

### 3.3 Mediating effect of social environment

Models 1a-1d estimated the relationship between the built environment and the mediating variables (social environment; Table 4).

The results showed that the distance to the nearest public transit station was significantly negatively correlated with social capital (*β* = −3.284, *p* < 0.05), while facility accessibility (*β* = <0.001, *p* < 0.1) was significantly positively correlated with social capital. Park accessibility was significantly positively correlated with social support (*β* = 0.113, p < 0.05). Mixed land use (*β* = 2.278, *p* < 0.01), facility accessibility (*β* = <0.001, *p* < 0.05), and distance to the nearest park (*β* = 0.226, *p* < 0.05) were significantly positively correlated with community safety. Public transit station accessibility (*β* = −0.013, *p* < 0.05) was significantly negatively correlated with community safety.

### 3.4 Relationship between the built environment, mediators, and mental health

Further, we added the mediating variables to the benchmark model for regression analysis to verify whether the mediating variables had a mediating effect on the mental health of older adults (Table 5).

**Table 5:** Mediation effect of the four mediators.

Variables	Modle2a (Mediator: Social capital)		Modle2b (Mediator: Social support)		Modle2c (Mediator: Community cohesion)		Modle2d (Mediator: Community safety)	
	Coef.	S. E	Coef.	S. E	Coef.	S. E	Coef.	S. E
Built environment								
Population density	0.033	0.227	0.078	0.231	0.027	0.227	0.012	0.226
Mixed land use	4.590	3.979	4.669	4.045	4.894	3.977	0.530	4.030
Facility accessibility	<0.001*	<0.001	<0.001	<0.001	<0.001**	<0.001	<0.001	<0.001
Park accessibility	−0.328	0.068	−0.021	0.069	−0.019	0.067	0.010	0.067
Public transit station accessibility	−0.063**	0.031	−0.074**	0.032	−0.081**	0.031	−0.055*	0.031
Distance to the nearest park	0.793	0.571	1.058*	0.579	0.786	0.571	0.794	0.570
Distance to the nearest public transit station	−0.729	1.519	−1.920	1.527	−1.134	1.508	−1.892	1.504
Mediator								
Social capital	0.411***	0.076						
Social support			0.061	0.075				
Community cohesion					1.181***	0.368		
Community safety							1.315***	0.220
Individual variables								
Age (ref.60–75)	−0.355	0.374	−0.542	0.378	−0.288	0.375	−0.493	0.371
Gender (ref. female)	0.399	0.437	0.378	0.444	0.280	0.437	0.376	0.436
Marital status (ref. unmarried)								
Widowed or divorced	−1.252	1.879	−1.429	1.913	−1.545	1.878	−1.957	1.874
Married	−1.132	1.838	−1.498	1.869	−1.637	1.836	−2.059	1.832
Education level (ref. primary school and below)								
Junior high school	0.597	0.458	0.435	0.464	0.534	0.456	0.707	0.457
Senior high school or technical secondary school	0.503	0.515	0.429	0.524	0.580	0.515	0.677	0.517
Junior college	0.006	0.922	−0.266	0.936	0.358	0.927	−0.017	0.917
University and above	0.428	1.196	0.177	1.215	0.408	1.195	−0.004	1.192
Monthly Income	0.816***	0.208	1.029***	0.208	0.927***	0.205	1.079***	0.203
Living arrangement (ref. living alone or with a spouse)								
Living with children	0.670*	0.368	0.600	0.374	0.697*	0.368	0.650*	0.367
Travel preferences (ref. walking or cycling)								
Public transportation	−0.979	1.001	−0.589	1.015	−0.811	0.998	−0.505	0.994
Self-driving or taking a taxi	0.408	0.439	0.574	0.445	0.509	0.437	0.830*	0.437
Constant	11.072**	3.601	17.296***	3.505	14.031***	3.441	15.629***	3.415
Log likelihood	−2626.810		−2640.801		−2626.087		−2613.682	
Within-group variance	<0.001		<0.001		<0.001		<0.001	
Between-group variance	5.096		5.179		5.091		5.072	
Prob > chi2	0.000		0.000		0.000		0.000	
AIC	5299.620		5237.601		5298.174		5273.364	

***, **, * significant at 1, 5, and 10% threshold level, respectively.

In Model 2a, facility accessibility (*β* = <0.001, *p* < 0.1) was significantly positively correlated with the mental health of older adults, while public transit station accessibility (*β* = −0.063, *p* < 0.05) was significantly negatively correlated. Social capital (*β* = 0.411, *p* < 0.01) was significantly positively correlated with the mental health of older adults.

In Model 2b, distance to the nearest park (*β* = 1.058, *p* < 0.1) was significantly positively correlated with the mental health of older adults, while public transit station accessibility (*β* = −0.074, *p* < 0.05) was significantly negatively correlated. Social support (*β* = 0.061, *p* > 0.1) showed no significant correlation with the mental health of older adults.

In Model 2c, facility accessibility (*β* = <0.001, *p* < 0.05) was significantly positively correlated with the mental health of older adults, while public transit station accessibility (*β* = −0.081, *p* < 0.05) was significantly negatively correlated. Community cohesion (*β* = 1.181, *p* < 0.01) was significantly positively correlated with the mental health of older adults.

In Model 2d, public transit station accessibility (*β* = −0.055, *p* < 0.1) was significantly negatively correlated with the mental health of older adults. Community safety (*β* = 1.315, *p* < 0.01) was significantly positively correlated with the mental health of older adults.

Further, we used Bootstrap to test the mediating effects of the mediating variables. Social capital played a full mediating role in the relationship between distance to the nearest public transit station and the mental health of older adults. Community safety played a full mediating role in the relationship between park accessibility and the mental health of older adults (Table 6).

**Table 6:** Results of the bootstrap test.

95% Confidence interval	Social capital	Community cohesion	Community safety
Park accessibility	(−0.007, 0.026)	(−0.017, 0.014)	(0.005, 0.036)
Distance to the nearest park	(−0.185, 0.071)	(−0.036, 0.200)	(−0.072, 0.145)
Distance to the nearest public transit station	(−0.959, −0.208)	(−0.471, 0.162)	(−0.304, 0.278)

### 3.5 Robustness test after avoiding self selection of residence

The mental health of older adults is influenced by the long-term effects of the built environment, and the choice of residential community directly determines the quality of the built environment. To minimize the potential impact of sampling randomness inherent in cross-sectional data, a robustness test was conducted by screening older adults who had relocated within the past 15 years.

Table 7 shows the associations between the built environment and the mental health of older adults with and without relocation experience. The results indicate differences in the associations between built environment and the mental health of these two groups. Population density, facility accessibility, and distance to the nearest park showed significant positive association with mental health of older adults with relocation experience. Public transit station accessibility and distance to the nearest public transit station showed significant negative association with mental health of older adults with relocation experience. The mental health of older adults without relocation experience was only positively influenced by the distance to the nearest park.

**Table 7:** Results of residential self-selection effects based on relocation experience.

Variables	Model 3a relocated		Model 3b not relocated	
	Coef.	S. E	Coef.	S. E
Population density	0.977**	0.440	−0.001	0.237
Mixed land use	3.284	4.613	1.616	3.622
Facility accessibility	1.556**	0.691	−0.324	0.307
Park accessibility	−0.137	0.127	0.027	0.065
Public transit station accessibility	−0.121**	0.059	−0.005	0.042
Distance to the nearest park	3.126*	1.772	0.428*	0.638
Distance to the nearest public transit station	−5.512	3.539	−1.156	1.801
AIC	1560.716		4170.423	
BIC	1638.276		4269.714	

S. E. Standard deviation, other covariates have been controlled in the model; ***, **, * means significant at 1, 5, and 10% levels, respectively.

### 3.6 Analysis of older adults groups with different income levels

Aside from the environment, income is also prominent among the various factors that may affect the mental health of older adults. Regardless of the social context in China or Western countries, there are significant differences in the living environments and mental health of older adults with different socioeconomic statuses. Furthermore, based on the median personal monthly income of the surveyed older adults (3,950 yuan), we divided the older adult population into two subsamples by income level: low income (≤ 3,950 RMB) and middle-to-high income (> 3,950 RMB). The intraclass correlation coefficient (ICC) for the null model of mental health among low-income older adults is 0.123, indicating that community factors explain 12.281% of the mental health variation in this income group. For middle-to-high-income older adults, the ICC of the null model for mental health is 0.201, meaning that community factors account for 20.097% of the mental health differences among middle-to-high-income older adults. These results justify the use of a multilevel regression model for analysis. Employing a methodology consistent with the previous research, we found that among low-income older adults, social capital plays a partial mediating role in the relationship between distance to the nearest park and mental health. Among middle-to-high-income older adults, community safety serves as a partial mediator in the relationship between distance to the nearest public transportation station and mental health. The results of this analysis are presented in the Appendix A (Table 8).

**Table 8:** Results of the bootstrap test (low-income and middle-to-high-income older adults).

Sub-group	Variables	Social capital	Social support	Community cohesion	Community safety
Low-income	Distance to the nearest park	(0.088, 0.091)	–	(−0.073, 0.101)	–
Middle-to-high-income	Distance to the nearest public transit station	(−0.959, 0.318)	–	(−0.844, 0.248)	(−0.234, −0.088)

## 4 Discussion

Previous empirical studies on the impact and pathways of the built environment on the health of older adults have mostly been conducted in the context of low-density cities in developed countries such as those in Europe and North America. Research in developing countries like China remains relatively scarce. This scarcity may lead to research conclusions that differ from those in Western developed countries due to variations in urban built environments, such as population density, road density, and public transportation systems. This study used Guangzhou as a representative high-density urban context in China to explore the impact of the built environment on the mental health of older adults and its pathways. This study confirms the positive role of parks in improving the mental health of older adults. More importantly, we found distance to the nearest public transit stations was significantly negatively correlated with the mental health of older adults.

**Figure 2:**
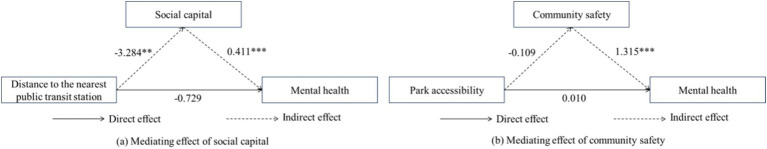
Path of mediating effect. **(a)** Path of mediating effect of social capital. **(b)** Path of mediating effect of community safety.

Being closer to public transit stations might actually weaken the social capital of older adults and further reduce their mental health levels (Figure 2a). This finding differs from conclusions drawn in developed countries. In megacities like Guangzhou, while population density increases and the transportation network becomes more developed, public transit stations located too close to communities can expose residents to noise and vehicle exhaust pollution (15). This increases the likelihood of older adults being exposed to noise pollution, dampens their enthusiasm for social interaction within the community, and is detrimental to mental health development (67, 68). Furthermore, long-term exposure to air pollution caused by vehicle exhaust is detrimental to mental health, and older adults are particularly susceptible populations to air pollution-related diseases (69) and related symptoms of depression and anxiety (70). Additionally, the convenience of public transport drives up the commercial value of surrounding areas. Traditional neighborhood shops (teahouses, chess rooms) are often replaced by convenience stores and fast-food outlets, depriving older adults of low-cost social gathering spots and reducing their corresponding social activities and social capital. This also reflects, to some extent, a deep-seated mismatch between urban spatial planning, community structural changes, and the needs of the older population in high-density urban contexts. When urban renewal focuses only on physical proximity and neglects the spatial rights of the older adults (right to safety, comfort, participation), the accessibility of public transit stations can instead become an accelerator of social exclusion.

Park accessibility may influence the mental health of older adults by affecting community safety (Figure 2b). Higher accessibility to parks may paradoxically be detrimental to the construction of a safe community environment and the mental well-being of older adults. The reason is that parks may provide hiding places for criminals and potentially conceal criminal activities (71), posing a threat and psychological pressure to older adults’ outdoor exercise activities, thereby being detrimental to mental health. Simultaneously, green spaces in highly urbanized areas can actually increase feelings of insecurity (72). This increased insecurity may be related to the poor maintenance level of green spaces in highly urbanized areas. The maintenance of green spaces is crucial for older adult’s sense of social safety. Graffiti, littering, vandalism, and other forms of disorder, more common in urban areas, can reduce sense of social safety (73). Although various indicators of parks in Guangzhou rank high nationally, there are still issues such as poor sanitation, chaotic management, and inadequate facilities. These problems may have affected older adults’ perception of community safety and their mental health levels to some extent.

For older adults with different income levels, among those with low income, there is a mediating pathway through social capital. Closer proximity to parks and can enhance the mental health of older adults by strengthening their social capital. Generally, in most cases, low-income older adults live in community with relatively poor conditions, placing them at a disadvantage in terms of access to public resources and social participation. This, to some extent, affects their ability to engage in health-improving activities. Therefore, for these low-income older adults groups, parks that provide public activity spaces are particularly important. Parks can increase the frequency of social interactions among older adults, expand their social networks, enhance their social capital, and facilitate the acquisition and dissemination of norms of healthy behavior. At the same time, the strong social capital of older adults enables them to connect with a few close friends and organize social activities in parks, thereby improving their mental health. Among middle-to-high-income older adults groups, there exists a mediating pathway through community safety. Closer proximity to the nearest public transit station may reduce community safety, thereby negatively impacting the mental health of this group. Generally, middle-to-high-income older adults reside in communities with relatively better surrounding environments, where nearby amenities and facilities may provide conditions for physical exercise and recreational activities. However, if the distance to public transit station is too short, it may introduce excessive pedestrian and vehicular traffic, increasing community safety concerns and affecting the sense of security when going out. This could, to some extent, reduce their motivation to engage in activities near the community, thereby hindering improvements in mental health. Additionally, middle-to-high-income older adults have a relative advantage in accessing public resources. Compared to low-income older adults groups, they tend to place greater emphasis on the experience and quality of accessing these resources, with community safety being one of the key factors they prioritize in this process.

This study has the following limitations. Firstly, the conclusions are based on cross-sectional data analysis, which may not fully reveal the causal relationship between the environment and the mental health of older adults. Secondly, the mediating and dependent variables used in this study were all self-reported subjective indicators by older adults. Future research could utilize instruments to obtain more objective data. Thirdly, the buffer zone is delineated with the community neighborhood committee as the center, which may cause some slight deviations in the results. In future research, we will add different delineation methods to improve the accuracy of the research results. Finally, as a typical representative of China’s high-density urban context, the findings of this study regarding Guangzhou require further validation for their generalizability and applicability to small and medium-sized cities as well as vast rural areas across China.

## 5 Conclusion

This study employed multi-level modeling and mediation effect analysis to examine the relationship between the built environment and the mental health of older adults, as well as the mediating pathways of the social environment between them. The results indicate that park accessibility and distance to the nearest park are significantly positively correlated with the mental health of older adults, while the distance to the nearest public transit station is significantly negatively correlated. Social capital and community safety play mediating roles in the relationships between park accessibility, distance to public transit stations, and the mental health of older adults, respectively. Furthermore, the impact of built environment on mental health of older adults through the social environment, as well as the mediating pathways involved, exhibits significant differences across older adults with varying income levels.

This study confirms the mediating role of the community social environment between the built environment and the health of older adults. Therefore, we recommend that urban planning and construction should address both “hardware” and “software” aspects. It is essential to build age-friendly physical spatial environments while also fully recognizing the importance of the social environment. The spatial planning and design of public transit stations should focus on expanding their functions from passing points to stopping points. (1) Design inclusive station squares and micro squares that can be planned. For example, near the exit of the station, plan a safety island or pocket square with priority for non motorized vehicles and pedestrians. Set up non slip and backrest rest seats (placed at intervals for easy conversation), and equip them with sunshade and rain proof awnings. This can become a natural gathering point for older adults to meet up for travel and pick up their grandchildren, creating a safe and comfortable stay space, transforming the surrounding area of the station from a hurried passing place to a social place, and encouraging older adults to engage in occasional social interactions. (2) Build embedded community services and social nodes. For example, planning a community living room or intergenerational center within the site complex or adjacent to the ground floor of the building. Operated by the government or social organizations, providing free tea, reading corners, chess and card tables, and regularly holding health lectures, smartphone training, anti fraud classes, etc. In addition, cooperation can be established with convenience stores and fast food restaurants to set up priority dining areas for older adults and community bulletin boards, in order to create a low-cost and easily accessible social hub, partially public commercial space, and promote the formation of information exchange and mutual assistance relationships among older adults. Transforming high accessibility park squares from potential risk spaces to safe healing spaces, thereby unleashing their positive psychological health potential. (1) Transparency transformation and visual field optimization. For example, trimming the dense shrubs on the edges of the park and both sides of the trail that obstruct the view, to ensure clear visibility from the external streets and the main activity areas inside. Remove or renovate walls and structures that create visual blind spots, enhance the possibility of natural monitoring, and allow older adults to feel visible and able to see clearly in the park, reducing their fear of unknown corners. (2) Layered lighting and security path. For example, implementing a safety lighting plan not only ensures bright main roads, but also provides targeted supplementary lighting for key nodes such as seating areas, toilet entrances, and parking lots. Soft warm white should be chosen for the lighting color temperature to avoid glare or heavy shadows. Ground embedded indicator lights can be set up to create a clear and safe way home. This significantly enhances the sense of security during nighttime and early morning/dusk periods, encouraging older adults to use the park more frequently.

## Data Availability

The data analyzed in this study is subject to the following licenses/restrictions: The data analyzed in this study is subject to the following licenses/restrictions the datasets presented in this article are not readily available because of institutional copyright issues. Requests to access these datasets should be directed to Zhang Rongrong, zhangrr94@zzuli.edu.cn.

## References

[1] Xu X Zhao Y Zhang X Xia S. Identifying the impacts of social, economic, and environmental factors on population aging in the Yangtze River Delta using the geographical detector technique. Sustainability. (2018) 10:1528. doi: 10.3390/su10051528

[2] Li D Li X Zeng Y. The moderating effect of community environment on the association between social support and Chinese older adults' health: an empirical analysis study. Front Public Health. (2022) 10:855310. doi: 10.3389/fpubh.2022.855310, 35570963 PMC9092342

[3] Wu L Huang Z Pan Z. The spatiality and driving forces of population ageing in China. PLoS One. (2021) 16:e0243559. doi: 10.1371/journal.pone.0243559, 33428682 PMC7799793

[4] Tang S Lee HF Feng J. Social capital, built environment and mental health: a comparison between the local elderly people and the ‘laopiao’ in urban China. Ageing Soc. (2020) 42:179–203. doi: 10.1017/s0144686x2000077x

[5] Zhang R Liu S Li M He X Zhou C. The effect of high-density built environments on elderly individuals' physical health: a cross-sectional study in Guangzhou, China. Int J Environ Res Public Health. (2021) 18:250. doi: 10.3390/ijerph181910250, 34639550 PMC8508494

[6] Zheng Z Chen H Yang L. Transfer of promotion effects on elderly health with age: from physical environment to interpersonal environment and social participation. Int J Environ Res Public Health. (2019) 16:794. doi: 10.3390/ijerph16152794, 31387307 PMC6696029

[7] Dendup T Astell-Burt T Feng X. Residential self-selection, perceived built environment and type 2 diabetes incidence: a longitudinal analysis of 36,224 middle to older age adults. Health Place. (2019) 58:102154. doi: 10.1016/j.healthplace.2019.102154, 31234122

[8] Firdaus G. Built environment and health outcomes: identification of contextual risk factors for mental well-being of older adults. Ageing Int. (2017) 42:62–77. doi: 10.1007/s12126-016-9276-0

[9] Hu H Cao Q Shi Z Lin W Jiang H Hou Y. Social support and depressive symptom disparity between urban and rural older adults in China. J Affect Disord. (2018) 237:104–11. doi: 10.1016/j.jad.2018.04.076, 29803900

[10] Domènech-Abella J Mundó J Leonardi M Chatterji S Tobiasz-Adamczyk B Koskinen S . Loneliness and depression among older European adults: the role of perceived neighborhood built environment. Health Place. (2020) 62:102280. doi: 10.1016/j.healthplace.2019.102280, 32479358

[11] Ram B Shankar A Nightingale CM Giles-Corti B Ellaway A Cooper AR . Comparisons of depression, anxiety, well-being, and perceptions of the built environment amongst adults seeking social, intermediate and market-rent accommodation in the former London Olympic athletes' village. Health Place. (2017) 48:31–9. doi: 10.1016/j.healthplace.2017.09.001, 28917115 PMC5711255

[12] Salvatore MA Grundy E. Area deprivation, perceived neighbourhood cohesion and mental health at older ages: a cross lagged analysis of UK longitudinal data. Health Place. (2021) 67:102470. doi: 10.1016/j.healthplace.2020.102470, 33212393

[13] Galea S Ahern J Rudenstine S Wallace Z Vlahov D. Urban built environment and depression: a multilevel analysis. J Epidemiol Community Health. (2005) 59:822–7. doi: 10.1136/jech.2005.033084, 16166352 PMC1732922

[14] Putrik P de Vries NK Mujakovic S van Amelsvoort L Kant I Kunst AE . Living environment matters: relationships between neighborhood characteristics and health of the residents in a Dutch municipality. J Community Health. (2015) 40:47–56. doi: 10.1007/s10900-014-9894-y, 24917124

[15] Zhang J Zheng Y Wen T Yang M Feng QM. The impact of built environment on physical activity and subjective well-being of urban residents: a study of core cities in the Yangtze River Delta survey. Front Psychol. (2022) 13:1050486. doi: 10.3389/fpsyg.2022.1050486, 36570995 PMC9773078

[16] Zhang L Zhou S Kwan MP. A comparative analysis of the impacts of objective versus subjective neighborhood environment on physical, mental, and social health. Health Place. (2019) 59:102170. doi: 10.1016/j.healthplace.2019.102170, 31422227

[17] Lei P Feng Z. Age-friendly neighbourhoods and depression among older people in China: evidence from China family panel studies. J Affect Disord. (2021) 286:187–96. doi: 10.1016/j.jad.2021.02.081, 33735763

[18] Brown SC Mason CA Lombard JL Martinez F Plater-Zyberk E Spokane AR . The relationship of built environment to perceived social support and psychological distress in Hispanic elders: the role of “eyes on the street”. J Gerontol. (2009) 64:234–46. doi: 10.1093/geronb/gbn011, 19196696 PMC2655159

[19] Kong X Han H Zhan M Chi F. The effects of the built environment on the mental health of older adults: a case study in Hangzhou, China. Innov Aging. (2024) 8:igae037. doi: 10.1093/geroni/igae037, 38707523 PMC11067799

[20] Mazumdar S Learnihan V Cochrane T Davey R. The built environment and social capital: a systematic review. Environ Behav. (2017) 50:001391651668734. doi: 10.1177/0013916516687343

[21] Zhu Y. Toward community engagement: can the built environment help? Grassroots participation and communal space in Chinese urban communities. Habitat Int. (2015) 46:44–53. doi: 10.1016/j.habitatint.2014.10.013, 26640314 PMC4665117

[22] Brown SC Lombard J. Neighborhoods and social interaction. Wellbeing. (2014) 1:1–28. doi: 10.1002/9781118539415.wbwell059

[23] Liu K Bearman PS. Focal points, endogenous processes, and exogenous shocks in the autism epidemic. Sociol Methods Res. (2015) 44:272–305. doi: 10.1177/0049124112460369, 26166907 PMC4495771

[24] Zhang R He X Liu Y Li M Zhou C. The relationship between built environment and mental health of older adults: mediating effects of perceptions of community cohesion and community safety and the moderating effect of income. Front Public Health. (2022) 10:1–15. doi: 10.3389/fpubh.2022.881169, 35784206 PMC9247295

[25] Cain CL Wallace SP Ponce NA. Helpfulness, trust, and safety of neighborhoods: social capital, household income, and self-reported health of older adults. Gerontologist. (2018) 58:4–14. doi: 10.1093/geront/gnx145, 29029195

[26] Lee YJ Braun KL Wu YY Hong S Gonzales E Wang Y . Neighborhood social cohesion and the health of native Hawaiian and other Pacific islander older adults. J Gerontol Soc Work. (2022) 65:3–23. doi: 10.1080/01634372.2021.1917033, 33974515

[27] Cramm JM Nieboer AP. Social cohesion and belonging predict the well-being of community-dwelling older people. BMC Geriatr. (2015) 15:30. doi: 10.1186/s12877-015-0027-y, 25879773 PMC4369354

[28] Brummett BH Mark DB Siegler IC Williams RB Babyak MA Clapp-Channing NE . Perceived social support as a predictor of mortality in coronary patients: effects of smoking, sedentary behavior, and depressive symptoms. Psychosom Med. (2005) 67:40–5. doi: 10.1097/01.psy.0000149257.74854.b7, 15673622

[29] Croezen S Picavet HS Haveman-Nies A Verschuren WM de Groot LC van’t Veer P. Do positive or negative experiences of social support relate to current and future health? Results from the Doetinchem cohort study. BMC Public Health. (2012) 12:65. doi: 10.1186/1471-2458-12-65, 22264236 PMC3275524

[30] Iwasaki Y Bartlett J O'Neil J. Coping with stress among aboriginal women and men with diabetes in Winnipeg, Canada. Soc Sci Med. (2005) 60:977–88. doi: 10.1016/j.socscimed.2004.06.032, 15589668

[31] Almedom AM. Social capital and mental health: an interdisciplinary review of primary evidence. Soc Sci Med. (2005) 61:943–64. doi: 10.1016/j.socscimed.2004.12.025, 15955397

[32] Kawachi I Kennedy BP Glass R. Social capital and self-rated health: a contextual analysis. Am J Public Health. (1999) 89:1187–93. doi: 10.2105/AJPH.89.8.1187, 10432904 PMC1508687

[33] Bierman A. Marital status as contingency for the effects of neighborhood disorder on older adults' mental health. J Gerontol B Psychol Sci Soc Sci. (2009) 64:425–34. doi: 10.1093/geronb/gbp010, 19251881 PMC2905133

[34] Wilson-Genderson M Pruchno R. Effects of neighborhood violence and perceptions of neighborhood safety on depressive symptoms of older adults. Soc Sci Med. (2013) 85:43–9. doi: 10.1016/j.socscimed.2013.02.028, 23540365

[35] Booth J Ayers S Marsiglia F. Perceived Neighborhood safety and psychological distress: exploring protective factors. J Sociol Soc Welfare. (2014) 2014:703. doi: 10.15453/0191-5096.3703

[36] Donder L Dury S De Witte N Verté D. Perceptual quality of neighbourhood design and feelings of unsafety. Ageing Soc. (2013) 33:207. doi: 10.1017/S0144686X12000207

[37] Qiu Y Liu Y Liu Y Li Z. Exploring the linkage between the Neighborhood environment and mental health in Guangzhou, China. Int J Environ Res Public Health. (2019) 16:3206. doi: 10.3390/ijerph16173206, 31480781 PMC6747328

[38] Tao Y Yang J Chai Y. The anatomy of health-supportive neighborhoods: a multilevel analysis of built environment, perceived disorder, social interaction and mental health in Beijing. Int J Environ Res Public Health. (2020) 17:13. doi: 10.3390/ijerph17010013, 31861358 PMC6981470

[39] Cao X Mokhtarian PL Handy SL. Do changes in neighborhood characteristics lead to changes in travel behavior? A structural equations modeling approach. Transportation. (2007) 34:535–56. doi: 10.1007/s11116-007-9132-x

[40] Mokhtarian PL Cao X. Examining the impacts of residential self-selection on travel behavior: a focus on methodologies. Transp Res B Methodol. (2008) 42:204–28. doi: 10.1016/j.trb.2007.07.006

[41] McCormack GR Shiell A. In search of causality: a systematic review of the relationship between the built environment and physical activity among adults. Int J Behav Nutr Phys Act. (2011) 8:125. doi: 10.1186/1479-5868-8-125, 22077952 PMC3306205

[42] Yanji Z Weitao D Lizhen Z Miaoyi LI. How urban built environment affects residents' physical health? Mediating mechanism and empirical test. Geogr Res. (2020) 39:822–35. doi: 10.11821/dlyj020190359

[43] Latpate R Kshirsagar J Gupta V Chandra G. Advanced sampling methods. Singapore: Springer (2021).

[44] Chunshan Z Tong X Wang J Lai S. Spatial differentiation and the formation mechanism of population aging in Guangzhou in 2000- 2010. Geogr Res. (2018) 37:103–18. doi: 10.11821/dlyj201801008

[45] Ware JE Sherbourne CD. The MOS 36-item short-form health survey (SF-36). i. Conceptual framework and item selection. Med Care. (1992) 30:473–83. 1593914

[46] Liu Y Wang R Xiao Y Huang B Chen H Li Z. Exploring the linkage between greenness exposure and depression among Chinese people: mediating roles of physical activity, stress and social cohesion and moderating role of urbanicity. Health Place. (2019) 58:102168. doi: 10.1016/j.healthplace.2019.102168, 31325813

[47] Zhou Y Yuan Y Chen Y Lai S. Association pathways between Neighborhood greenspaces and the physical and mental health of older adults-a cross-sectional study in Guangzhou, China. Front Public Health. (2020) 8:551453. doi: 10.3389/fpubh.2020.551453, 33072696 PMC7536577

[48] Chen Y Yuan Y. The neighborhood effect of exposure to blue space on elderly individuals' mental health: a case study in Guangzhou, China. Health Place. (2020) 63:102348. doi: 10.1016/j.healthplace.2020.102348, 32543435

[49] Elliott LR White MP Taylor AH Herbert S. Energy expenditure on recreational visits to different natural environments. Soc Sci Med. (2015) 139:53–60. doi: 10.1016/j.socscimed.2015.06.038, 26151390

[50] Robert SA. Socioeconomic position and health: the independent contribution of community socioeconomic context. Annu Rev Sociol. (1999) 25:489–516. doi: 10.1146/annurev.soc.25.1.489

[51] Ewing R Cervero R. Travel and the built environment. J Am Plan Assoc. (2010) 76:265–94. doi: 10.1080/01944361003766766

[52] Pearson AL Bentham G Day P Kingham S. Associations between neighbourhood environmental characteristics and obesity and related behaviours among adult new Zealanders. BMC Public Health. (2014) 14:553. doi: 10.1186/1471-2458-14-553, 24894572 PMC4059100

[53] Saelens BE Handy SL. Built environment correlates of walking: a review. Med Sci Sports Exerc. (2008) 40:S550–66. doi: 10.1249/MSS.0b013e31817c67a4, 18562973 PMC2921187

[54] Zhong Y Schön P Burström B Burström K. Association between social capital and health-related quality of life among left behind and not left behind older people in rural China. BMC Geriatr. (2017) 17:287. doi: 10.1186/s12877-017-0679-x, 29246251 PMC5732484

[55] Jiang C Chow JC Zhou L Song H Shi J. Community support, social isolation and older adults' life satisfaction: evidence from a national survey in China. Aging Ment Health. (2024) 28:849–57. doi: 10.1080/13607863.2023.2277871, 37921357

[56] Weimann H Rylander L van den Bosch MA Albin M Skärbäck E Grahn P . Perception of safety is a prerequisite for the association between neighbourhood green qualities and physical activity: results from a cross-sectional study in Sweden. Health Place. (2017) 45:124–30. doi: 10.1016/j.healthplace.2017.03.011, 28359908

[57] Baron RM Kenny DA. The moderator-mediator variable distinction in social psychological research: conceptual, strategic, and statistical considerations. J Pers Soc Psychol. (1986) 51:1173–82. doi: 10.1037//0022-3514.51.6.1173, 3806354

[58] Lu X White H. Robustness checks and robustness tests in applied economics. J Econom. (2014) 178:194–206. doi: 10.1016/j.jeconom.2013.08.016

[59] Zhou J Wang G Huang Z. Does productivity improvement affect rural human capital accumulation? A micro-perspective study. Econ Res. (2019) 54:15.

[60] Ju X Zhao X Sun B. Internet and trade costs: an empirical analysis based on cross-border E-commerce data from China SME exports. Econ Res. (2020) 55:15.

[61] Yang R. Industrial agglomeration and regional wage gap: an empirical study based on 269 cities in China. Manag World. (2013) 8:11. doi: 10.19744/j.cnki.11-1235/f.2017.07.007

[62] Sun C Luo Y Yao X. The effects of transportation infrastructure on air quality: evidence from empirical analysis in China. Econ Res. (2019) 54:15.

[63] Artamonova A Gillespie BJ Brandén M. Geographic mobility among older people and their adult children: the role of parents' health issues and family ties. Popul Space Place. (2020) 26:e2371. doi: 10.1002/psp.2371, 33935604 PMC8072412

[64] Wilmoth JM. Health trajectories among older movers. J Aging Health. (2010) 22:862–81. doi: 10.1177/0898264310375985, 20710006

[65] Stoeckel K Porell F. Do older adults anticipate relocating? The relationship between housing relocation expectations and falls. J Appl Gerontol. (2010) 29:231–50. doi: 10.1177/0733464809335595

[66] Mulder CH Kooiman N. Moving for proximity to family, care needs and the locations of family members: an analysis of matched survey and register data. Popul Space Place. (2024) 30:e2713. doi: 10.1002/psp.2713

[67] Coan JA Sbarra DA. Social baseline theory: the social regulation of risk and effort. Curr Opin Psychol. (2015) 1:87–91. doi: 10.1016/j.copsyc.2014.12.021, 25825706 PMC4375548

[68] Holt-Lunstad J. Why social relationships are important for physical health: a systems approach to understanding and modifying risk and protection. Annu Rev Psychol. (2018) 69:437–58. doi: 10.1146/annurev-psych-122216-011902, 29035688

[69] Sacks Jason D Wichers Stanek Lindsay J Luben Thomas O Johns Douglas J Buckley Barbara S James B . Particulate matter–induced health effects: who is susceptible? Environ Health Perspect. (2011) 119:446–54. doi: 10.1289/ehp.1002255 20961824 PMC3080924

[70] Pun VC Manjourides J Suh H. Association of Ambient air Pollution with depressive and anxiety symptoms in older adults: results from the NSHAP study. Environ Health Perspect. (2017) 125:342–8. doi: 10.1289/ehp494, 27517877 PMC5332196

[71] Andrews M Gatersleben B. Variations in perceptions of danger, fear and preference in a simulated natural environment. J Environ Psychol. (2010) 30:473–81. doi: 10.1016/j.jenvp.2010.04.001

[72] Maas J Spreeuwenberg P van Winsum-Westra M Verheij RA Vries S Groenewegen PP. Is green space in the living environment associated with people's feelings of social safety? Environ Plann A. (2009) 41:1763–77. doi: 10.1068/a4196

[73] Burgess J Harrison CM Limb M. People, parks and the urban green: a study of popular meanings and values for open spaces in the city. Urban Stud. (1988) 25:455–73. doi: 10.1080/00420988820080631

