# Supplementary material for: Exploring the mediating role of social environment in the relationship between built environment and mental health of older adults—evidence from Guangzhou, China
Source: Front Public Health. 2026 Jan 26;14:1706431. doi: 10.3389/fpubh.2026.1706431 (PMC12884539; doi:10.3389/fpubh.2026.1706431)
Supplement: Supplementary file 1 [file Table_1.docx]

**Appendix A**

Table 9 The Direct Effects of Built Environment on Mental Health of Older Adults with Different Income Levels (Low-income and Middle-to-High-income Groups)

|  | Model 3-1 and 3-2  (DV: Mental health) | | | Model 3-1a and 3-2a  (DV: Social capital) | | Model 3-1b and 3-2b  (DV: Social support) | | Model 3-1c and 3-2c  (DV: Community cohesion) | | Model 3-1d and 3-2d  (DV: Community safety) | |
| --- | --- | --- | --- | --- | --- | --- | --- | --- | --- | --- | --- |
|  | S.E.  (Low-income) | | S.E.  (Middle-to-High-income) | S.E.  (Low-income) | S.E.  (Middle-to-High-income) | S.E.  (Low-income) | S.E.  (Middle-to-High-income) | S.E.  (Low-income) | S.E.  (Middle-to-High-income) | S.E.  (Low-income) | S.E.  (Middle-to-High-income) |
| **Built Environment** |  | |  |  |  |  |  |  |  |  |  |
| Population density | -0.421 | | 0.856 | -0.268 | 0.164 | -0.243* | -0.285** | -0.039 | 0.027 | -0.007 | 0.116** |
| Mixed land use | -17.516 | | -0.947 | 1.415 | 2.824 | 1.684 | 2.188 | 0.646 | 0.718 | 1.327** | 0.894 |
| Facility accessibility | 1.836 | | 0.233 | -0.260 | -0.338 | -0.327** | -0.259 | -0.043 | -0.209* | 0.075 | 0.048 |
| Park accessibility | 0.568 | | 0.014 | 0.010 | 0.028 | 0.122** | 0.051 | -0.012 | -0.013 | 0.017 | -0.003 |
| Public transit station accessibility | -0.185 | | -0.125 | 0.026 | -0.017 | 0.044** | 0.037 | 0.008 | 0.014 | -0.007 | -0.004 |
| Distance to the nearest park | 4.695* | | 2.758 | 1.010* | 0.461 | 0.304 | 0.214 | 0.472*** | -0.006 | 0.354** | 0.338* |
| Distance to the nearest public transit station | -12.426 | | -12.827** | -4.001** | -1.485 | -0.085 | 0.862 | -1.175** | -0.258 | -0.646 | -0.070 |
| Constant | 31.817 | | 38.127 | 21.002 | 21.171 | 13.653 | 9.440 | 3.684 | 3.509 | 2.382 | 3.357 |
| Log likelihood | -2473.805 | -1686.289 | | -1089.904 | -1075.929 | -1083.432 | -1097.667 | -572.591 | -575.985 | -568.586 | -567.067 |
| Prob > chi2 | 0.757 | 0.000 | | 0.005 | 0.006 | 0.002 | 0.053 | 0.000 | 0.003 | 0.005 | 0.001 |
| AIC | 4987.610 | 3414.58 | | 2219.808 | 2193.858 | 2206.863 | 2237.334 | 1185.182 | 1193.970 | 1177.172 | 1176.135 |

Other covariates have been controlled for in the model，***, **, * significant at 1%, 5%, and 10% threshold level, respectively.

Table 10 The Mediating Effects of Built Environment on Mental Health of Older Adults with Different Income Levels (Low-income and Middle-to-High-income Groups)

|  | Model 4-1a and 4-2a  (Mediator: Social capital) | | Model 4-1b and 4-2b  (Mediator: Social support) | | Model 4-1c and 4-2c (Mediator: Community cohesion) | | | Model 4-1d and 4-2d  (Mediator: Community safety) | |
| --- | --- | --- | --- | --- | --- | --- | --- | --- | --- |
|  | S.E.  (Low-income) | S.E.  (Middle-to-High-income) | S.E.  (Low-income) | S.E.  (Middle-to-High-income) | S.E.  (Low-income) | S.E.  (Middle-to-High-income) | | S.E.  (Low-income) | S.E.  (Middle-to-High-income) |
| **Built Environment** |  |  |  |  |  |  | |  |  |
| Population density | -0.376 | 0.815 | -0.349 | 0.868 | -0.451 | 0.832 | | -0.422 | 0.707 |
| Mixed land use | -17.745 | -1.957 | -18.011 | -1.040 | -17.039 | -1.620 | | -18.929 | -1.844 |
| Facility accessibility | 1.892 | 0.354 | 1.933 | 0.237 | 1.805 | 0.432 | | 1.802 | 0.222 |
| Park accessibility | 0.564 | 0.006 | 0.532 | 0.012 | 0.559 | 0.026 | | 0.532 | 0.015 |
| Public transit station accessibility | -0.191 | -0.123 | -0.198 | -0.127 | -0.180 | -0.135 | | -0.177 | -0.121 |
| Distance to the nearest park | 3.495* | 2.505 | 3.601 | 2.724 | 4.043 | 2.759 | | 3.146 | 2.571 |
| Distance to the nearest public transit station | -11.622 | -12.359* | -12.401 | -12.853** | -13.293 | -12.584** | | -12.210 | -12.829** |
| **Mediator** |  |  |  |  |  |  | |  |  |
| Social capital | 0.196* | 0.379** |  |  |  |  | |  |  |
| Social support |  |  | 0.293 | 0.039 |  |  | |  |  |
| Community cohesion |  |  |  |  | 0.737** | 0.952** | |  |  |
| Community safety |  |  |  |  |  |  | | 0.983 | 1.169** |
| Constant | 27.632 | 30.347 | 27.812 | 37.816 | 34.534 | 34.791 | | 29.344 | 33.565 |
| Log likelihood | -2473.775 | -1683.596 | -2473.740 | -1686.258 | -2473.760 | | -1684.199 | -2454.521 | -1683.234 |
| Within-group variance | <0.001 | 2.079 | <0.001 | 1.991 | <0.001 | | 1.974 | <0.001 | 1.839 |
| Between-group variance | 4.099 | 8.061 | 5.056 | 8.113 | 4.299 | | 8.079 | 4.110 | 8.074 |
| Prob > chi2 | 0.806 | 0.000 | 0.802 | 0.001 | 1.000 | | 0.000 | 1.000 | 0.000 |
| AIC | 4989.55 | 3411.192 | 4989.481 | 3416.515 | 4989.512 | | 3412.399 | 4951.042 |  |

Other covariates have been controlled for in the model，***, **, * significant at 1%, 5%, and 10% threshold level, respectively.
